# Antecedents of Individuals’ Concerns Regarding Hospital Hygiene and Surgery Postponement During the COVID-19 Pandemic: Cross-sectional, Web-Based Survey Study

**DOI:** 10.2196/24804

**Published:** 2021-03-11

**Authors:** Thomas Ostermann, Julia Gampe, Jan Philipp Röer, Theda Radtke

**Affiliations:** 1 Department of Psychology and Psychotherapy Witten/Herdecke University Witten Germany; 2 Health Psychology and Applied Diagnostics University of Wuppertal Wuppertal Germany

**Keywords:** COVID-19, public health, medical investigations, surgery, hospitalization, medical practices

## Abstract

**Background:**

The COVID-19 pandemic poses a major challenge to people’s everyday lives. In the context of hospitalization, the pandemic is expected to have a strong influence on affective reactions and preventive behaviors. Research is needed to develop evidence-driven strategies for coping with the challenges of the pandemic. Therefore, this survey study investigates the effects that personality traits, risk-taking behaviors, and anxiety have on medical service–related affective reactions and anticipated behaviors during the COVID-19 pandemic.

**Objective:**

The aim of this study was to identify key factors that are associated with individuals’ concerns about hygiene in hospitals and the postponement of surgeries.

**Methods:**

We conducted a cross-sectional, web-based survey of 929 residents in Germany (women: 792/929, 85.3%; age: mean 35.2 years, SD 12.9 years). Hypotheses were tested by conducting a saturated path analysis.

**Results:**

We found that anxiety had a direct effect on people’s concerns about safety (β=−.12, 95% CI −.20 to −.05) and hygiene in hospitals (β=.16, 95% CI .08 to .23). Risk-taking behaviors and personality traits were not associated with concerns about safety and hygiene in hospitals or anticipated behaviors.

**Conclusions:**

Our findings suggest that distinct interventions and information campaigns are not necessary for individuals with different personality traits or different levels of risk-taking behavior. However, we recommend that health care workers should carefully address anxiety when interacting with patients.

## Introduction

In Germany, the first COVID-19 case was confirmed at the end of January 2020, and COVID-19 incidence rates rose in the following 3 months. In response, the Robert Koch Institute (ie, the German federal government agency and research institute responsible for disease control and prevention) and the Federal Centre for Health Education made the following recommendations to slow the interpersonal transmission of SARS-CoV-2: limit social contact, refrain from traveling unless absolutely necessary, work from home wherever possible, encourage the use of medical masks and gloves, and strengthen hand hygiene practices [1]. At the same time, the European Center for Disease Prevention and Control published a checklist to prepare hospitals for the reception and care of patients with COVID-19. This checklist included items that were related to hand hygiene, personal protective equipment, and the postponement of operations that were unrelated to COVID-19 [2]. However, the implementation of these regulations, particularly those regarding the use of personal protective equipment during the initial weeks of the pandemic in Germany, was hindered by a lack of adequate medical masks and clothing [3]. Considering the fact that SARS-CoV-2 infection can result in severe illness and death, especially in people aged >65 years and those with defined risk factors (eg, high blood pressure, diabetes, chronic respiratory diseases, and cancer) [4], a lack of personal protective equipment in hospitals and inadequate medical practices can result in affective reactions (eg, worries and concerns) and anticipated behaviors (eg, the denial of important operations) among the general population [5,6].

An example of an affective reaction resulting from a concern about an impending or anticipated threat is worrying about the lack of personal protective equipment in hospitals. Various factors, such as sociodemographic characteristics and personal values, can be used to predict affective reactions [5,7]. Further, affective reactions like concern or worry positively relate to anxiety [8] and negatively relate to risk-taking behaviors [9]. In addition, personality traits (eg, neuroticism) are linked with affective reactions [10]. During the COVID-19 pandemic, it is necessary to investigate the possible antecedents of affective reactions that relate to hospital equipment and medical practices. Such information is necessary for training health care workers to develop psychological skills for helping patients who experience worry, anxiety, and other emotional problems. It is also necessary to investigate how the COVID-19 pandemic affects people’s reactions when they or a person close to them needs to be hospitalized to undergo surgery for treating an illness [11,12]. Studies have shown that the COVID-19 pandemic poses a considerable challenge to routine medical services. For example, a study reported that patients prefer to postpone their operations until after the pandemic has completely passed due to the uncertain environment [12]. However, none of the studies that have been conducted during the pandemic have investigated psychological concepts that might influence individuals’ concerns about hospital hygiene and the postponement of surgeries. Studies on treatment-related decisions have suggested that personality traits, risk-taking behaviors, and anxiety are important factors that affect people’s decisions to avoid visiting a hospital or doctor [13,14].

Based on previous pandemics, it is known that segmenting the population into subgroups (ie, sociodemographic subgroups) is important for designing and delivering messages about health risks and health protection measures [15,16]. However, even though this might be a useful and effective method, these subgroups do not account for several important psychological factors, such as personality traits or anxiety. These factors might be crucial antecedents of affective reactions to public health messages. They might also influence people’s health-related decisions. Specifically, these factors may directly affect anticipated behaviors that relate to people’s decisions to postpone a nonurgent surgery [12,17]. Therefore, this survey study aims to identify the key factors that are associated with hospitalization-related and medical service–related affective reactions and anticipated behaviors during the COVID-19 pandemic.

We hypothesized that individuals with low levels of openness, high levels of conscientiousness, low levels of extraversion, low levels of agreeableness, high levels of neuroticism, low levels of risk-taking behavior, and high levels of anxiety would experience high levels of negative affective reactions and exhibit high levels of anticipated preventive behaviors in response to hospitalization and medical service provision.

## Methods

### Survey Summary

This cross-sectional, web-based survey study took place between March 19 and April 17, 2020. To ensure that our survey was highly visible to potential respondents, it was distributed via social media, email, direct communication methods, and advertisements in various digital communication channels. The recruitment of participants mainly took place at the Department of Psychology of Witten/Herdecke University. All participants were residents of Germany who were aged ≥16 years. All procedures in this study were performed in accordance with the ethical standards of the institutional review board of the Department of Psychology and Psychotherapy of Witten/Herdecke University and those of the American Psychology Association [18,19]. A letter of approval can be obtained from the first author.

### Measures

#### Summary of Survey Instruments

Prior to the survey, we screened potentially eligible test instruments and scales to assess their suitability for answering the hypotheses. We selected validated scales (ie, whenever possible) for measuring the different survey constructs. We also developed new scales to measure the COVID-19–specific aspects of the survey, as no validated instruments were available at the time of the survey. The development of survey items was based on existing scales from other behavioral domains.

The following survey items, which were answered by using a visual analog scale that ranged from 0 (ie, not at all) to 100 (ie, absolutely), served as dependent variables: affective reactions and anticipated behaviors.

#### Affective Reactions

Affective reactions [20] were measured with two items for assessing concerns about hospital safety, hospital hygiene, and medical practices during the COVID-19 pandemic. After providing a short introduction to place the questions in the context of the COVID-19 pandemic, the following questions were asked: (1) “recently there have been supply bottlenecks of mouthguards, disinfectants or similar for hospitals and medical practices. Do you feel safe in places like this”; and (2) “how big is your concern that due to supply bottlenecks a proper hygiene cannot be ensured in hospitals or medical practices?”

#### Anticipated Behaviors

Anticipated behaviors were measured with two items for assessing people’s decisions to postpone their own surgery or advise a person close to them against surgery during the pandemic. These items were in line with previous studies [11,12]. After providing a short introduction to place the questions in the context of the COVID-19 pandemic, the following questions were asked: (1) “assuming you were about to have a non-urgent surgery - how likely would you be to postpone this surgery”; and (2) “suppose a person very close to you was about to have a non-urgent surgery, how likely is it that you would advise against having the surgery?”

The following survey items served as independent variables: personality, risk-taking behaviors, and anxiety.

#### Personality

People’s personalities were measured with the Big Five Inventory (BFI)-10, which is the short version of the BFI-44 [21]. The BFI assesses the following five personality traits: openness to experience, conscientiousness, extraversion, agreeableness, and neuroticism (OCEAN). Openness to experience refers to whether people are inventive/curious or consistent/cautious. Conscientiousness refers to whether people are efficient/organized or easygoing/careless. Extraversion refers to whether people are outgoing/energetic or solitary/reserved. Agreeableness refers to whether people are friendly/compassionate or challenging/detached. Neuroticism refers to whether people are sensitive/nervous or secure/confident. Our psychometric analyses indicated that BFI-10 scores sufficiently correlated with BFI-44 scores. Based on the average correlation value (*r*=0.83), 70% of the variance in BFI-44 scores could be explained. After 6-8 weeks, the BFI-10 had an average retest reliability value of 0.75.

#### Risk-Taking Behaviors

Risk-taking behaviors were assessed with the readiness to take risk/search for competition scale of the Hamburger Personality Inventory (HPI), which includes 14 items that are evaluated with a 4-point Likert scale (eg, “Ultimately, I am also unstoppable by massive threats”). HPI item scores are added to calculate a risk-taking score [22]. With a Cronbach α value of .85, the HPI has high content and construct validity. The HPI has a test-retest reliability value of 0.86 after 18 months. Additionally, HPI scores positively correlate with autonomy orientations (*r*=0.48), revolutionary tendencies (*r*=0.53), conflict skills (*r*=0.53), and competitive attitudes (*r*=0.60). These scores also negatively correlate with harm avoidance tendencies (*r*=−0.78).

#### Anxiety

Anxiety was measured with the German version of the Spielberger State-Trait Anxiety Inventory (STAI), which is one of the most commonly used standard tools for measuring anxiety. In research, STAI scores also function as an indicator of distress. The state anxiety portion of the STAI consists of 20 items that are evaluated on a 4-point Likert scale (eg, “I feel worried”). All item scores are added to calculate a state anxiety score [23]. Higher STAI scores indicate greater anxiety/distress. The STAI has Cronbach α values that range between .90 and .94, which means that it has high content and construct validity. According to the original publication [23], the test-retest reliability coefficients of the STAI range between 0.65 to 0.75 (ie, within 2 months of completing the STAI). These coefficients remained stable in our psychometric analyses.

To assess whether people’s risk of contracting COVID-19 and information-seeking behaviors (ie, those related to COVID-19) had an impact on their worries and anticipated behaviors, the following constructs were included in our analysis as covariates: risk profile and information-seeking behaviors.

#### Risk Profile

Risk profiles were adapted in accordance with previous studies [4,24]. Our survey included seven dichotomous items (ie, yes=1; no=0) that asked about risk factors for contracting COVID-19 (ie, age of >60 years, chronic lung disease, autoimmune disease, diabetes, kidney or liver diseases, cancer, immune deficiency, and the intake of immunosuppressive remedies). The sum of the item scores was used as a risk profile.

#### Information-Seeking Behaviors

Information-seeking behaviors were adapted in accordance with a previous study [25]. The behaviors we analyzed were in line with another study [26]. Our survey included six dichotomous items (ie, yes=1; no=0) that asked about the sources that people used to obtain information on COVID-19 (ie, television, internet blogs, social media, the website of the German federal government agency that is responsible for disease control and prevention, newspapers, and tabloid press articles). The sum of the item scores was used as an indicator of information-seeking behavior intensity. In addition, age, gender, and educational level (ie, a dichotomous variable that accounted for primary and secondary education) were introduced in the path model as covariates that needed to be controlled.

### Statistical Analysis Strategy

**Figure 1:**
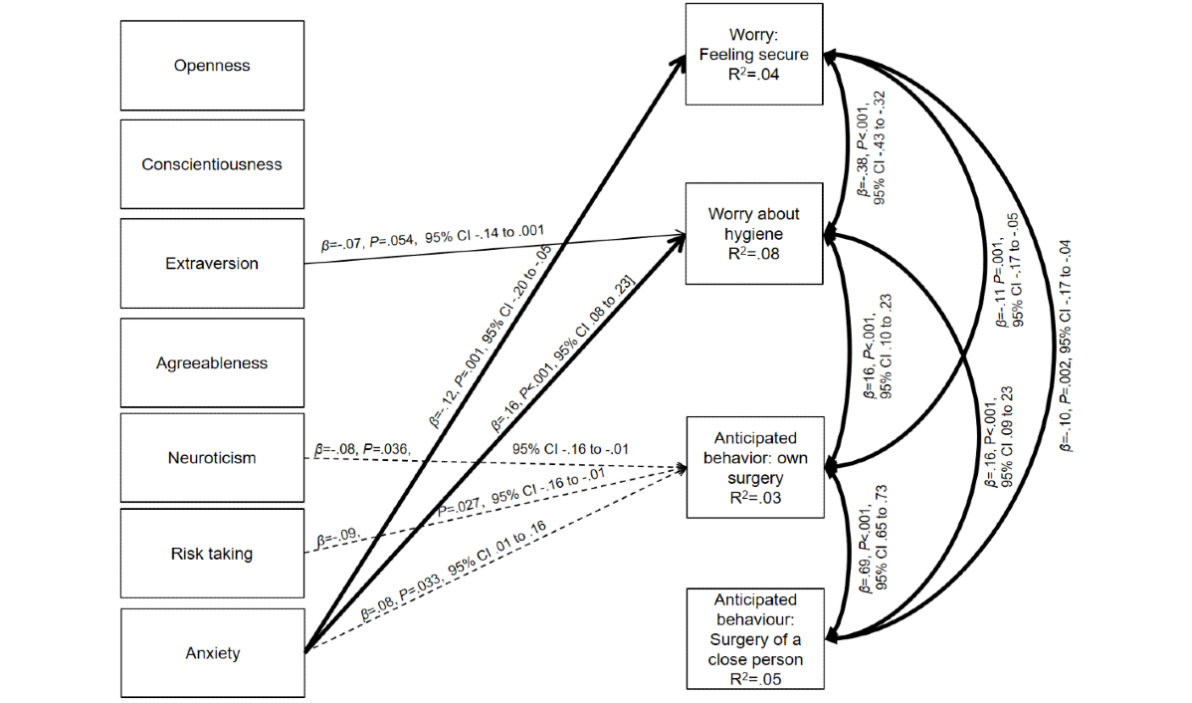
The hypothesized path model for identifying associations between independent variables (ie, personality traits, risk-taking behaviors, and anxiety) and dependent variables (ie, worries about safety, worries about hygiene, and anticipated behaviors). The model used data from 929 participants. We did not display the control variables (ie, risk profiles, information-seeking behaviors, age, gender, and education) to keep the model overview simple. Dotted lines refer to *P* values of ≥.01 and ≤.05. Bold lines refer to *P* values of <.001. Thin lines refer to *P* values of ≥.05. We did not display correlations between the control variables and outcomes to keep the model overview simple.

Participants who fully completed the questionnaires were included in the statistical analysis. Descriptive statistical analyses were performed to describe the sample’s characteristics in terms of the variables that were included in this study. In addition, bivariate correlation values were computed to examine associations among the variables. A saturated path model [27] with manifest variables was used to test whether OCEAN personality traits, anxiety, and risk-taking behaviors were related to worries about hospital safety and hygiene, worries about medical practices, and anticipated behaviors toward nonurgent surgeries (Figure 1). To assess whether people’s risk of contracting COVID-19 and information-seeking behaviors (ie, those related to COVID-19) had an impact on their worries and anticipated behaviors, these variables were included in the analysis.

Age, gender, and educational level (ie, a dichotomous variable that accounted for primary and secondary education) were introduced in the model as covariates that needed to be controlled. All variables in the model were allowed to covary. Standardized regression coefficients (ie, β_i_) for the path model (ie, the model for predicting affective reactions) and anticipated behaviors were calculated with the decomposition equation of correlations (ie, *r*_i_), which is used to determine the direct and indirect effects that predictor variables (ie, X_i_) have on dependent variables (ie, Y_j_). In addition, the 95% CIs were calculated based on the 2.5 and 97.5 percentiles of the estimated SEs from bootstrapping [27]. As our study had four main dependent variables, Bonferroni correction was performed to lower the Cronbach α level for interpreting the results (ie, from .05 to .0125). To evaluate model fitness, the Chi-square test was used. According to Bollen and Long [28], Chi-square values should not be 2-5 times larger than the degrees of freedom. In addition, comparative fit index, Tucker-Lewis index, root mean square error of approximation, and standardized root mean square residual values were calculated as fit indices. Descriptive statistical analyses were conducted with SPSS, version 26 (IBM Corporation). The path analysis was conducted with Mplus, version 8.1 (Muthén & Muthén) [29].

## Results

### Descriptive Characteristics

Of the 1059 participants who took part in our survey, 929 (87.7%) had complete data sets. Thus, these 929 participants were included in the analyses. As indicated in Table 1, most of the participants (792/929, 85.3%) were female. The mean age of participants was 35.3 years (SD 12.9 years). Of the 929 participants, 890 (95.8%) stated that they were not infected with SARS-CoV-2, and only 7 (0.8%) stated that they were infected with SARS-CoV-2 (ie, at the time of the survey or before the survey). With respect to risk profiles, 683 (73.5%) participants reported that they did not exhibit any of the risk factors for contracting COVID-19, while 112 (12.1%) stated that they had a chronic lung disease. Almost all participants (884/929, 95.2%) subjectively felt restricted due to COVID-19–related regulations and measures. Details on participants’ sociodemographic characteristics are provided in Table 1.

Descriptive statistics and correlations among the variables in the path model are reported in Tables 2 and 3. Worries about proper hospital hygiene and medical practices positively correlated with neuroticism (*r*=0.12) and anxiety (*r*=0.21). Further, all four dependent variables intercorrelated with each other. For example, worries about hygiene and worries about safety significantly correlated with each other (*r*=−0.40; *P*=.001).

**Table 1:** The sample’s sociodemographic characteristics.

Sociodemographic variables		Value	
Age (years), mean (SD)		35.3 (12.9)	
Age (years), median (range)		32 (16-82)	
**Sex, n (%)**			
	Male		137 (14.7)
	Female		792 (85.3)
**Educational level, n (%)**			
	No school degree		3 (0.3)
	Secondary school		6 (1.7)
	Secondary modern education		108 (11.6)
	Vocational baccalaureate		75 (8.1)
	General baccalaureate		223 (24)
	Applied science university diploma		116 (12.5)
	Bachelor’s degree		181 (19.5)
	Master’s degree		172 (18.5)
	Doctorate degree or higher		34 (3.7)
**COVID-19 status, n (%)**			
	Not infected		890 (95.8)
	I was under suspicion		17 (1.8)
	I am under suspicion		15 (1.6)
	I was infected		5 (0.5)
	I am infected		2 (0.2)
**Risk profile, n (%)**			
	No risk factors		683 (73.5)
	Aged >60 years		50 (5.4)
	Chronic lung disease		112 (12.1)
	Autoimmune disease		66 (7.1)
	Diabetes		31 (3.3)
	Cancer		15 (1.6)
	Immunodeficiency		56 (6)
	Intake of immunosuppressants		43 (4.6)
**Information source, n (%)**			
	Friends and family		369 (39.7)
	Television		553 (59.5)
	Internet in general		401 (43.2)
	Social media		402 (43.3)
	Dedicated websites		758 (81.6)
	Newspapers		495 (53.3)
	Tabloid press articles		29 (3.1)
**Feeling restricted due to COVID-19–related regulations and measures, n (%)**			
	Yes		884 (95.2)
	No		45 (4.8)

**Table 2:** Bivariate correlations (ie, r values) among variables.

Variable			Conscientiousness		Extraversion		Agreeableness		Neuroticism		RTB^a^		Anxiety		Risk profile		Information profile		Feeling secure^b^		Hygiene^b^		Own surgery^c^		Surgery of a close person^c^
**Openness**																									
	*r*	0.03		0.03		0.03		−0.01		0.12^e^		0.01		0.02		−0.07^e^		0.01		0.03		0.01		<−0.01	
	*P* value	.39		.10		.35		.77		<.001		0.7		.57		0.05		.78		.38		.73		.91	
**Conscientiousness**																									
	*r*	—^d^		0.12^e^		0.08^e^		−0.15^e^		0.12^e^		−0.12^e^		0.03		−0.08^e^		0.02		−0.04		0.02		0.06	
	*P* value	—		<.001		.02		<.001		<.001		<.001		.38		.02		.59		.25		.51		.09	
**Extraversion**																									
	*r*	—		—		0.13^e^		−0.30^e^		0.22^e^		−0.22^e^		−0.01		<0.01		0.05		−0.11^e^		−0.01		−0.02	
	*P* value	—		—		<.001		<.001		<.001		<.001		.75		.90		.13		<.001		.88		.57	
**Agreeableness**																									
	*r*	—		—		—		−0.12^e^		−0.04		−0.19^e^		−0.03		0.01		0.08^e^		-0.09^e^		−0.02		<−0.01	
	*P* value	—		—		—		<.001		.23		<.001		.41		.84		.02		.007		.47		.95	
**Neuroticism**																									
	*r*	—		—		—		—		−0.32^e^		0.48^e^		0.03		0.03		−0.05		0.13^e^		−0.01		0.01	
	*P* value	—		—		—		—		<.001		<.001		.36		.43		.11		<.001		.69		.79	
**RTB**																									
	*r*	—		—		—		—		—		−0.20^e^		0.04		−0.04		0.04		−0.05		−0.09^e^		−0.06	
	*P* value	—		—		—		—		—		<.001		.24		.23		.19		.14		.007		.07	
**Anxiety**																									
	*r*	—		—		—		—		—		—		0.09^e^		0.07^e^		−0.14^e^		0.21^e^		0.08^e^		0.06	
	*P* value	—		—		—		—		—		—		.005		.04		<.001		<.001		.02		.05	
**Risk profile**																									
	*r*	—		—		—		—		—		—		—		0.06		−0.07^e^		0.12^e^		0.02		0.07^e^	
	*P* value	—		—		—		—		—		—		—		.08		.03		<.001		.47		.04	
**Information profile**																									
	*r*	—		—		—		—		—		—		—		—		<−0.01		0.04		−0.01		0.05	
	*P* value	—		—		—		—		—		—		—		—		.98		.25		.77		.16	
**Feeling secure^b^**																									
	*r*	—		—		—		—		—		—		—		—		—		−0.40^e^		−0.13^e^		−0.13^e^	
	*P* value	—		—		—		—		—		—		—		—		—		<.001		<.001		<.001	
**Hygiene^b^**																									
	*r*	—		—		—		—		—		—		—		—		—		—		0.18^e^		0.18^e^	
	*P* value	—		—		—		—		—		—		—		—		—		—		<.001		<.001	
**Own surgery^c^**																									
	*r*	—		—		—		—		—		—		—		—		—		—		—		0.70^e^	
	*P* value	—		—		—		—		—		—		—		—		—		—		—		<.001	

^a^RTB: risk-taking behavior.

^b^Refers to a worry.

^c^Refers to an anticipated behavior category.

^d^Not applicable.

^e^Significant at a level of *P*<.05.

**Table 3:** Mean and SD values of variables.

Variable	Value, mean (SD)
Openness	7.60 (2.02)
Conscientiousness	7.15 (1.65)
Extraversion	6.66 (1.97)
Agreeableness	6.20 (1.58)
Neuroticism	6.26 (2.04)
Risk-taking behavior	31.26 (7.13)
Anxiety	43.91 (12.23)
Risk profile	0.40 (0.80)
Information profile	3.24 (1.36)
Worries about feeling secure	48.32 (28.23)
Worries about hygiene	57.33 (30.35)
Anticipated behavior relating to own surgery	80.46 (28.45)
Anticipated behavior relating to the surgery of a close person	77.32 (28.93)

### Results From the Path Model

The path model predicted the associations between independent variables (ie, personality, risk-taking behaviors, and anxiety) and dependent variables (ie, feelings about security, worries about hospital hygiene and medical practices, and anticipated behaviors that relate to people’s decisions to postpone their own surgery or advise a person close to them against surgery). Figure 1 presents the parameter estimates of the model (ie, standardized solutions).

The following model-data fit indices were obtained: Chi-square value (*χ*^2^_54_=942.94; N=929; *P*<.001), comparative fit index (1.00), Tucker-Lewis Index (1.00), root mean square error of approximation (<.01), and standardized root mean square residual (<.01). These values indicated a moderate model fitness. Table 4 provides the standardized regression coefficients of the path model, which was used to predict affective reactions and anticipated behaviors.

**Table 4:** Standardized regression coefficients of the path model, which was used to predict affective reactions and anticipated behaviors.

Path predictors	Affective reactions		Anticipated behaviors	
	Feeling secure, β (95% CI)	Concerns about hygiene, β (95% CI)	Own surgery, β (95% CI)	Surgery of a close person, β (95% CI)
Openness	<.01 (−.07 to .08)	.02 (−.05 to .09)	.02 (−.04 to .09)	−.02 (−.09 to .05)
Conscientiousness	.01 (−.05 to .08)	−.03^a^ (−.09 to .04)	.01 (−.06 to .08)	.03 (−.03 to .10)
Extraversion	.03 (−.04 to .10)	−.07^a^ (−.14 to <.01)	.01 (−.06 to .08)	−.02 (−.09 to .05)
Agreeableness	.05 (−.02 to .12)	−.05^a^ (−.11 to .02)	−.03 (−.10 to .05)	<.01 (−.06 to .07)
Neuroticism	.05 (−.03 to .13)	.01 (−.07 to .09)	−.08^b^ (−.16 to −.01)	−.02 (−.10 to .06)
Risk-taking behavior	.01 (−.07 to .08)	<.01 (−.07 to .08)	−.09^b^ (−.16 to −.01)	−.04 (−.12 to .03)
Anxiety	−.12^c^ (−.20 to −.05)	.16^d^ (.08 to .23)	.08^b^ (.01 to .16)	.05 (−.03 to .13)
Risk profile	−.06 (−.14 to .01)	.08^b^ (.02 to .14)	<.01 (−.07 to .08)	.01 (−.05 to .08)
Information-seeking behavior	−.01 (−.09 to .07)	−.01 (−.07 to .05)	.01 (−.14 to .15)	.03 (−.05 to .10)
Gender^e^	.09^b^ (.02 to .17)	−.06^a^ (−.12 to .01)	−.08^a^ (−.16 to .01)	−.08^b^ (−.15 to −.01)
Age	−.05 (−.13 to .03)	.10^c^ (.03 to .17)	.09^b^ (.01 to .16)	.18^d^ (.11 to .25)
Education^f^	.03 (−.05 to .11)	−.08^b^ (−.16 to <−.01)	−.06 (−.16 to .04)	−.11^c^ (−.19 to −.03)

^a^Significant at a level of *P*<.10.

^b^Significant at a level of *P*<.05.

^c^Significant at a level of *P*<.001.

^d^Significant at a level of *P*<.001.

^e^In the path model, women were given a value of 1 and men were given a value of 2.

^f^In the path model, secondary education was given a value of 1 and tertiary education was given a value of 2.

As outlined in Figure 1, the feeling of security with regard to hospitals and medical practices was significantly negatively related to anxiety (β=−.12; *P*=.001), which is in line with our hypothesis. Further, affective reactions to hospital hygiene and medical practices resulting from a bottleneck of appropriate personal protective equipment for health care workers were significantly positively related to anxiety (β=.16; *P*<.001) and nonsignificantly negatively related to extraversion (β=−.07; *P*=.054). Although anticipated behaviors that relate to advising a close person against surgery did not correlate with any of our hypothesized variables, anticipated behaviors that relate to one’s own surgery were negatively associated with neuroticism (β=−.08; *P*=.04) and risk-taking behaviors (β=−.09; *P*=.03). Such anticipated behaviors were also positively associated with anxiety (β=.08; *P*=.03). All of these associations however were not statistically significant after the Bonferroni correction. No other associations between the independent and dependent variables were found. However, women and older participants reported that they experienced higher levels of negative affective reactions and anticipated behaviors compared to men and younger participants, respectively.

## Discussion

### Principal Findings

To the best of our knowledge, our study is the first to investigate predictors of affective reactions that relate to hospital safety, hospital hygiene, and medical practices during the COVID-19 pandemic. We are also the first to investigate anticipated behaviors that relate to people’s decisions to postpone their surgery or advise a person close to them against surgery during the pandemic. Our findings are in line with those of a German-Austrian survey [30], which found that anxiety was positively related to security actions. Our results suggest that state anxiety is the most influential factor of anticipated health-related behaviors and concerns about safety or hygiene. Apart from state anxiety, none of the other hypothesized predictors (eg, risk-taking behaviors) or personality factors (eg, agreeableness or openness) had any significant association with affective reactions or anticipated behaviors. This is contradictory to the recent findings of Martin [31], who found that agreeableness was related to the perceived severity of the COVID-19 pandemic, and openness was related to low levels of anxiety with regard to contracting COVID-19. Although previous studies have suggested that individuals with high levels of neuroticism exhibit pronounced negative reactions to stressful events [32], our findings show that neuroticism was not associated with anticipated behaviors during the COVID-19 pandemic. In our study, we found that people with high levels of neuroticism were less likely to postpone their own surgery. This finding is comparable to that of an early US survey, which found that neuroticism was associated with high levels of concern [33]. The similarities in these results could be explained by the age of our participants. It is possible that our relatively young participants were not able to accurately imagine a scenario in which they are hospitalized. This might have influenced participants’ responses to our survey. With regard to the relationship between health policy formation and public responses [34], the most important finding of our study was that anxiety was related to both the affective reactions and anticipated behaviors of the participants. Allgleton and Kippax [35], who conducted an analysis on Australian HIV/AIDS policies, argued that suppressed anxiety can be used as a depressive position for eliciting a desired response in the general public [35]. Other studies [36-38] have also found that anxiety is an important predictive factor of taking preventive measures and exhibiting compliant behavioral responses during the 2009 influenza pandemic. Our findings support these empirical results. Health authorities should be aware that anxiety may not only affect individuals’ behaviors but also the behaviors of organizations and systems (eg, splitting and blaming) [39]. In addition, anxiety resulting from the COVID-19 pandemic might also encourage individuals to consult a physician later than necessary (ie, to present their complaints). This has already occurred [40]. Such behavior may result in harms to health, the development of depression [41], or the chronification of disease.

### Limitations and Implications for Future Research

Aside from the strengths of our study (eg, its large sample size), several limitations also need to be mentioned. First, due to the dynamic nature of the pandemic, we decided to use a random sample. However, due to our survey dissemination methods, our sample may not be representative of the German population. The generalizability of our results is open to empirical debate, as our sample mostly consisted of middle-aged and well-educated women. Research has shown that compared to men, women are more likely to actively seek health-related information and pay more attention to potential worldwide pandemics [42]. Second, our sample mainly consisted of middle-aged individuals. Therefore, it is reasonable to assume that older people are more likely to postpone surgeries and operations due to the COVID-19 pandemic, as they are more susceptible to the disease than middle-aged people. Older people also have stronger health care needs than middle-aged people [17]. Further research on the COVID-19–related concerns of older individuals is needed. Third, data collection took place during the beginning of the pandemic in Germany. Therefore, it remains unclear whether individuals would have the same affective reactions and anticipated behaviors later into the pandemic. Furthermore, the affective reactions and anticipated behaviors of people from urban areas should be distinguished from those of people from rural areas, as COVID-19 spreads at different rates in different geographical areas [43]. However, during the first phase of the pandemic in Germany, no considerable differences were found in infection and death rates [43]. This finding is also supported by the results of a recent survey study [44], wherein the authors did not find any substantial differences in behavioral intentions between participants from rural and urban regions in China. Fourth, our dependent variables were only measured with one item that used a visual analog scale. This was done to keep the survey concise and specific. Unfortunately, validated measures such as the COVID-19–Induced Anxiety Scale or the Protective Behaviors Towards COVID-19 Scale [45] were not available at the time of our survey. According to Heller et al [46] and Price et al [47], visual analog scales have sufficient psychometric measurement properties. Thus, they can be used when no validated instrument is available. Fifth, although the fitness of our path model was acceptable, it could have been better. However, it should be noted that as the sample size increases and the degrees of freedom remain constant, the Chi-square value increases. This leads to the problem of plausible models being rejected due to a significant Chi-square value. Therefore, too much emphasis should not be placed on the significance of the Chi-square statistic [48]. Furthermore, it should be noted that our data are cross-sectional in nature. As such, causal conclusions cannot be drawn from our data. Future studies should be longitudinal in nature.

### Conclusions

Our results provide further insight into affective reactions and anticipated health-related behaviors during the COVID-19 pandemic. Our findings indicate that OCEAN personality traits are not associated with affective reactions and anticipated behaviors. Therefore, specific distinctions do not seem necessary when designing messages about health risks and health protection measures (ie, those related to hospital and medical practices during the COVID-19 pandemic). Even though future research is needed to confirm our results, health care workers should address the issues of patients with anxiety seriously and directly. Clear communication is necessary when providing information on the specific actions that hospitals and medical organizations perform to protect patients and health care workers. This could also help with preventing the cancellation of nonurgent surgeries in hospitals.

## Abbreviations

BFI: Big Five Inventory
HPI: Hamburger Personality Inventory
OCEAN: openness to experience, conscientiousness, extraversion, agreeableness, and neuroticism
STAI: State-Trait Anxiety Inventory
